# Large Salt Dust Storms Follow a 30-Year Rainfall Cycle in the Mar Chiquita Lake (Córdoba, Argentina)

**DOI:** 10.1371/journal.pone.0156672

**Published:** 2016-06-03

**Authors:** Enrique H. Bucher, Ariel F. Stein

**Affiliations:** 1 Instituto de Diversidad y Ecología Animal (CONICET-UNC) and Centro de Zoología Aplicada, Facultad de Ciencias Exactas Físicas y Naturales, Universidad Nacional de Córdoba, Rondeau 798, 5000 Córdoba, Argentina; 2 NOAA / Air Resources Laboratory, R/ARL—NCWCP—Room 4205, 5830 University Research Court, College Park, Maryland 20740, United States of America; University of Vigo, SPAIN

## Abstract

Starting in 2006, a new source of intense salt dust storms developed in Mar Chiquita (Córdoba, Argentina), the largest saline lake in South America. Storms originate from vast mudflats left by a 30-year expansion-retreat cycle of the lake due to changes in the regional rainfall regime. The annual frequency of salt dust storms correlated with the size of the salt mudflats. Events were restricted to the coldest months, and reached up to 800 km from the source. Occurrence of dust storms was associated with specific surface colors and textures easily identifiable in satellite images. High-emission surfaces were characterized by the presence of sodium sulfate hydrous/anhydrous crystals (mirabilite and thenardite), and a superficial and variable water table, which may result in the periodic development of a characteristic “fluffy” surface derived from salt precipitation-dissolution processes. HYSPLIT model simulation estimates a deposition maximum near the sources (of about 2.5 kg/ha/yr), and a decreasing trend from the emission area outwards, except for the relative secondary maximum modeled over the mountain ranges in southern Bolivia and northern Argentina due to an orographic effect. The 2009 total deposition of salt dust generated in Mar Chiquita was estimated at 6.5 million tons.

## Introduction

Desiccation of salt lakes and the resulting increase of exposed dry lake beds are creating a rapidly growing source of salt dust around the world. This process is sometimes related to changes in the rainfall regime, but often to human intervention. Salt dust storms (SDS onwards) are major actors in the world's arid and semi-arid regions, not only because usually they are an important sign of desertification and land degradation, but also because they produce a whole suite of important environmental impacts. Compared with common dust storms, SDS transport high concentrations of fine-grain saline and alkaline material, such as sodium sulfate, sodium chloride, and other potentially toxic components [1].

A recent and very spectacular example of this trend is the case of the Aral Sea, where large-scale development of irrigation projects led to a reduced water discharge of the rivers flowing into the lake, which in turn caused the accelerated desiccation of much of the lake surface, and the subsequent generation of large SDS [2, 3]. Similarly, many other lakes have been affected by this process in arid zones around the world, including Owens Lake and Salton Sea in the USA, and Aiby Lake and Ebinur Lake in China [1, 4]. In Argentina, the Mar Chiquita lake (one of the largest salt lakes in the world) has been mentioned as a dust source by [4, 5, 6].

Starting in 2005, a new situation emerged in Mar Chiquita, in terms of the start of frequent and massive SDS events never recorded before [5]. These SDS had clear distinctive characteristics, particularly in terms of their origin and intensity.

In the first place, the observed SDS originated not from the lake shrinking due to water diversion in the upper tributaries, but instead from a marked cycle of level rise and decline within a period of about 30 years, which resulted in vast saline playas from where the SDS generated [5]. From these new playas, frequent and significant SDS started to be generated, with dust plumes of over 500 km long and 90 km wide, comparable with those recorded in the well-known case of the Aral Sea [2, 3].

Here we describe a) the characteristics, frequency, and dimension of the salt dust emission in Mar Chiquita during the 2004–2014 period, and b) estimate the geographical area and amount of salt deposition resulting from the recorded events during this period.

## Materials and Methods

### Regional background

**Fig 1:**
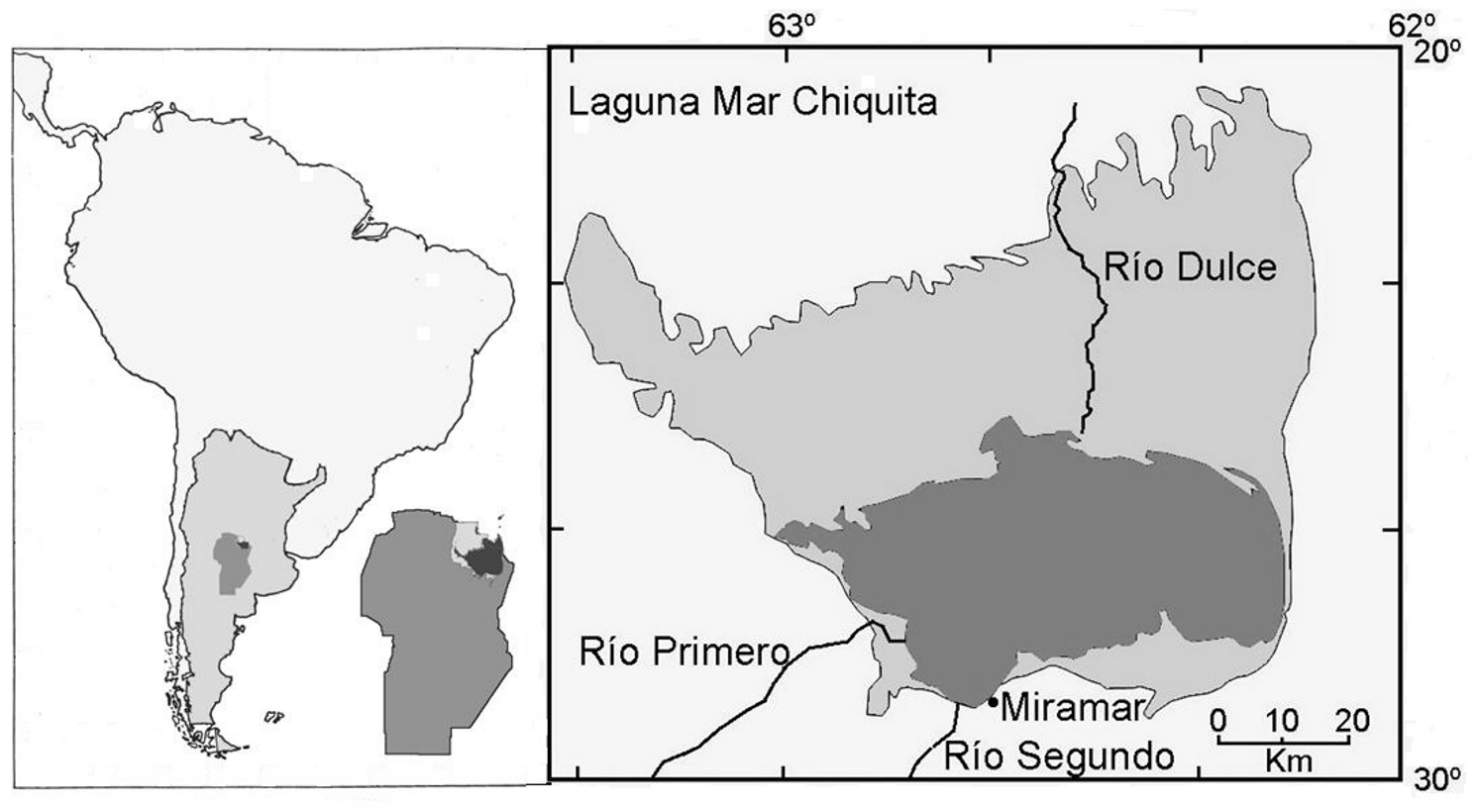
The Mar Chiquita Lake. Left: situation of the Mar Chiquita Lake in South America, Argentina, and Córdoba province. Right: Detailed map of Mar Chiquita. Dark gray: original area. Light gray: area of expansion in the 1974–2004 period.

Mar Chiquita is a shallow, saline lake of tectonic origin located in the vast plains of central Argentina, at an elevation of about 62 m above sea level (a.s.l.). It is the final collector of a huge basin of about 40,000 km^2^ of mostly flat relief with mountain ranges on the west. The lake is permanent, with an area that has fluctuated historically between 2000 and 6000 km^2^ [5], (Fig 1).

Three main tributaries flow into the lake: the Primero, Segundo, and Dulce rivers (Fig 1). The Dulce River has the largest basin and provides about 80% of the incoming water. It comes from the North of Mar Chiquita, and in its final portion flows along a wide floodplain of a low slope gradient, covered by saline wetlands with halophytic vegetation maintained by periodic flooding and fire. The Primero and Segundo rivers come from the south and provide the remaining 20% of the incoming water flow (Fig 1). The original vegetation around the lake was mainly semi-arid savannas and woodlands, which has been largely replaced by agriculture.

The Mar Chiquita area holds a rich biodiversity, particularly in birds. It is a provincial Reserve, a Ramsar site of the Convention on Wetlands (known as the Ramsar Convention), and a site of Hemispheric Importance for the Western Hemisphere Shorebird Reserve Network (WHSRN) [5].

#### Climate and hydrology

**Fig 2:**
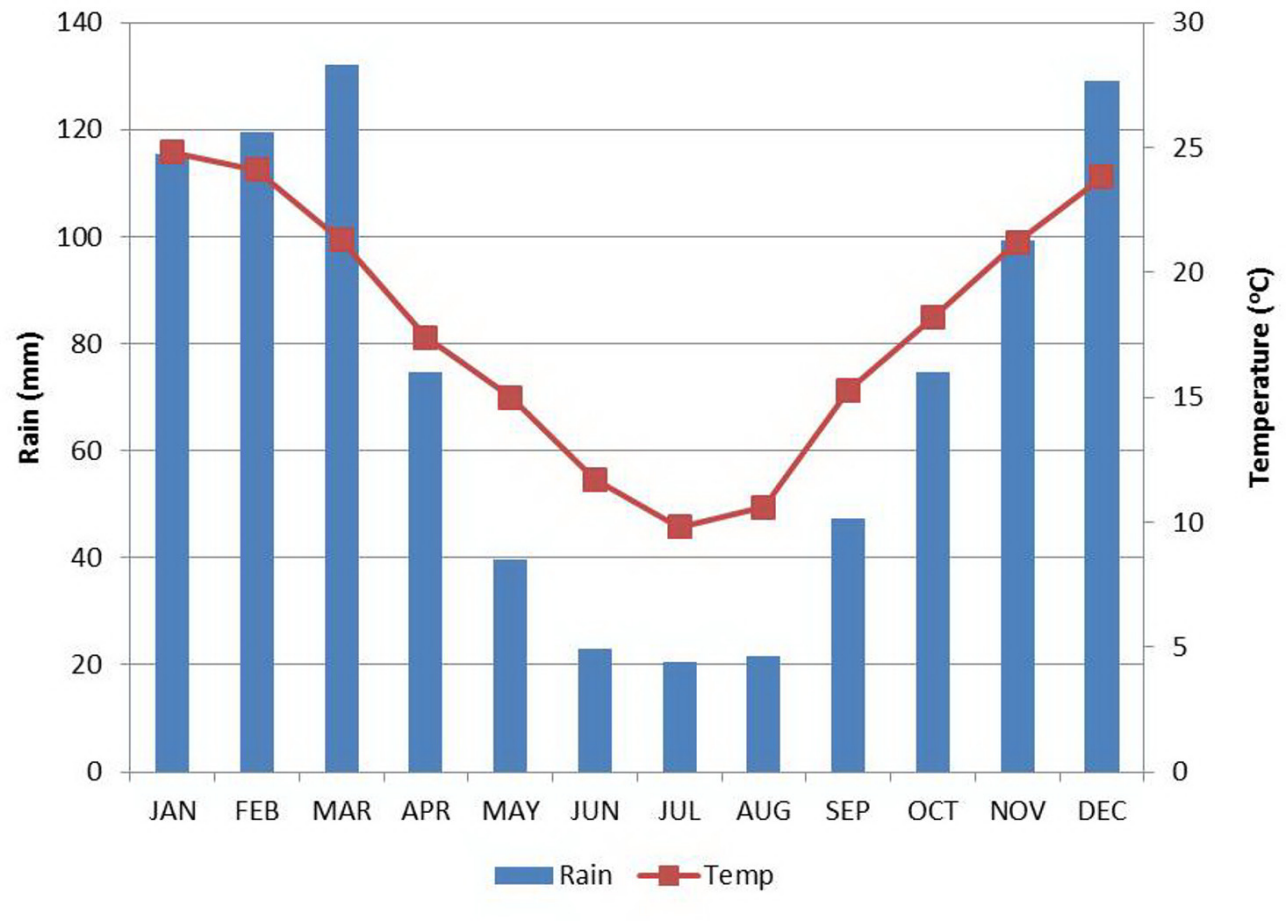
Average monthly temperature and precipitation in Mar Chiquita. Bars: monthly rainfall; line: monthly mean temperature. Annual rainfall: 878 mm; Mean annual temperature 18.1°C. Locality: Miramar (30° 57’S, 56° 20’ W, elevation 78 m asl). Servicio Meteorologico Nacional, Argentina.

Mar Chiquita is in a subtropical, semiarid region (Fig 2). Mean annual rainfall ranges between 800 and 900 mm, concentrated in the summer months (October to March). Mean annual temperature is 19°C, with a mean maximum of the warmest month (January) of 32°C, and a mean minimum of the coldest month (July) of 4.5°C. The annual evapotranspiration rate has been estimated at 1,448 mm for the 1966–1996 period, corrected for water salinity. Accordingly, the regional climate is characterized by the marked excess of potential evapotranspiration over precipitation. Therefore, the lake level results from the rather unstable balance between rainfall and water inflow versus evaporation, since ground seepage does not appear significant [5, 6].

#### Water chemistry

Water chemistry of the Mar Chiquita Lake is characterized by predominance of sodium chloride (78%), sodium sulfate (17%), calcium sulfate (2%) and magnesium sulfate (2%) (values indicate proportion of total salt content, in weight). According to the lake level, salinity has ranged between 300 g/L and 25 g/L. Sodium chloride and sodium sulphate are also predominant in evaporites in salt playas [7].

#### Origin of the large salt playas

Since the beginning of the 20th century Mar Chiquita has gone through marked variations in water level and surface area, which have been key in developing SDS. These changes include four identifiable periods. During the first period, between 1900–1972, the level was low, ranging between 68 m and 62 m a.s.l. Maximum depth was between 2 and 3 m, with most of the lake area being very shallow. The second period (1972–1981) was characterized by a rapid and substantial increase in water level (of about 8 m) that ended at 71 m a.s.l. [7].

During the third period (1981–2003) the lake level remained relatively stable at a high level, reaching an absolute peak in 2003 (71.79 m a.s.l.), the highest in the lake geological history [8, 9]. A vast portion of the Dulce River flood plain was invaded by the waters, and all terrestrial and marsh vegetation in the flooded area was eliminated.

**Fig 3:**
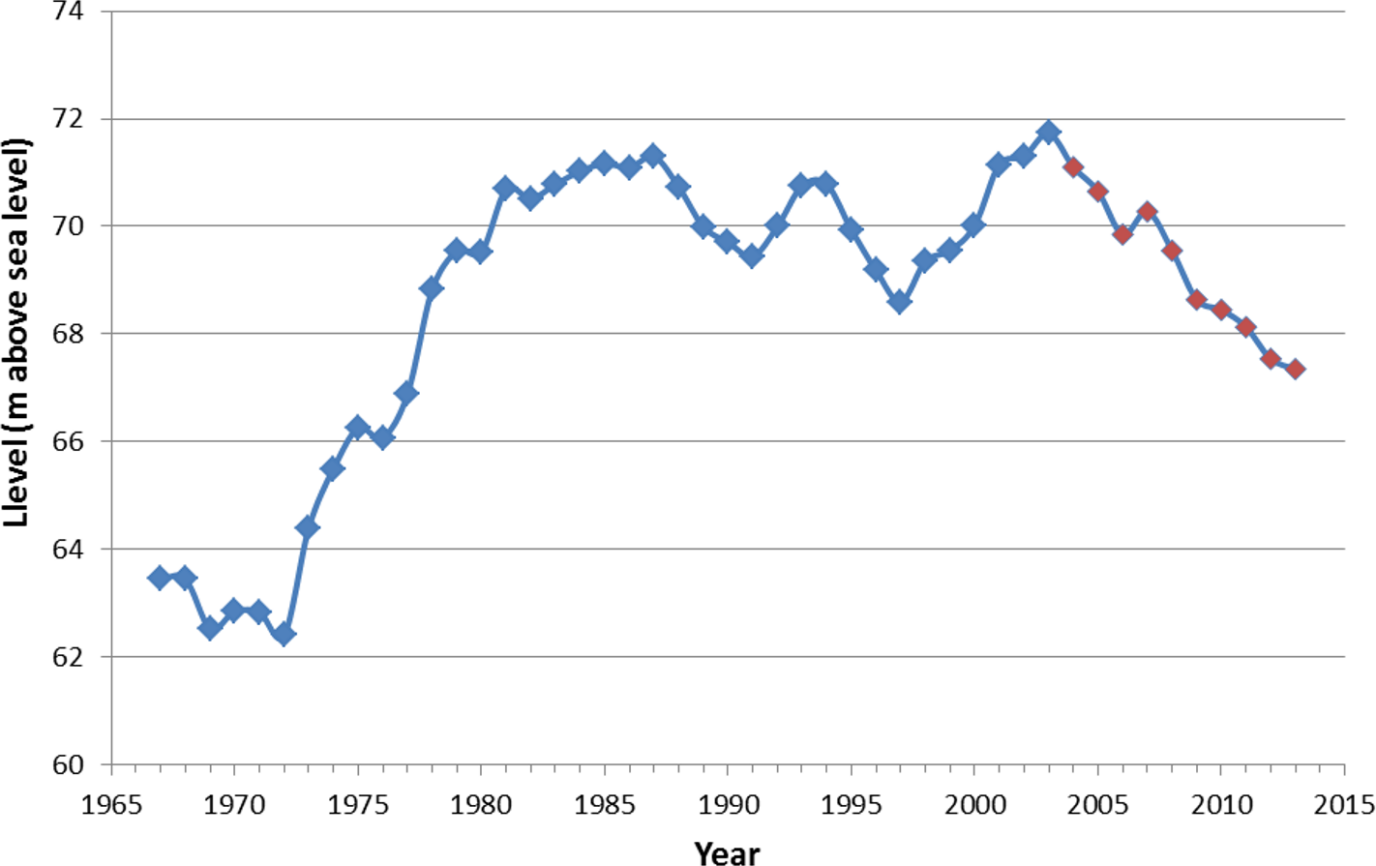
Water level of Mar Chiquita Lake in the 1967–2013 period. In red, years when dust storms were recorded. Data from the Mar Chiquita Field Station, PROMAR, Universidad Nacional de Córdoba, Argentina.

**Fig 4:**
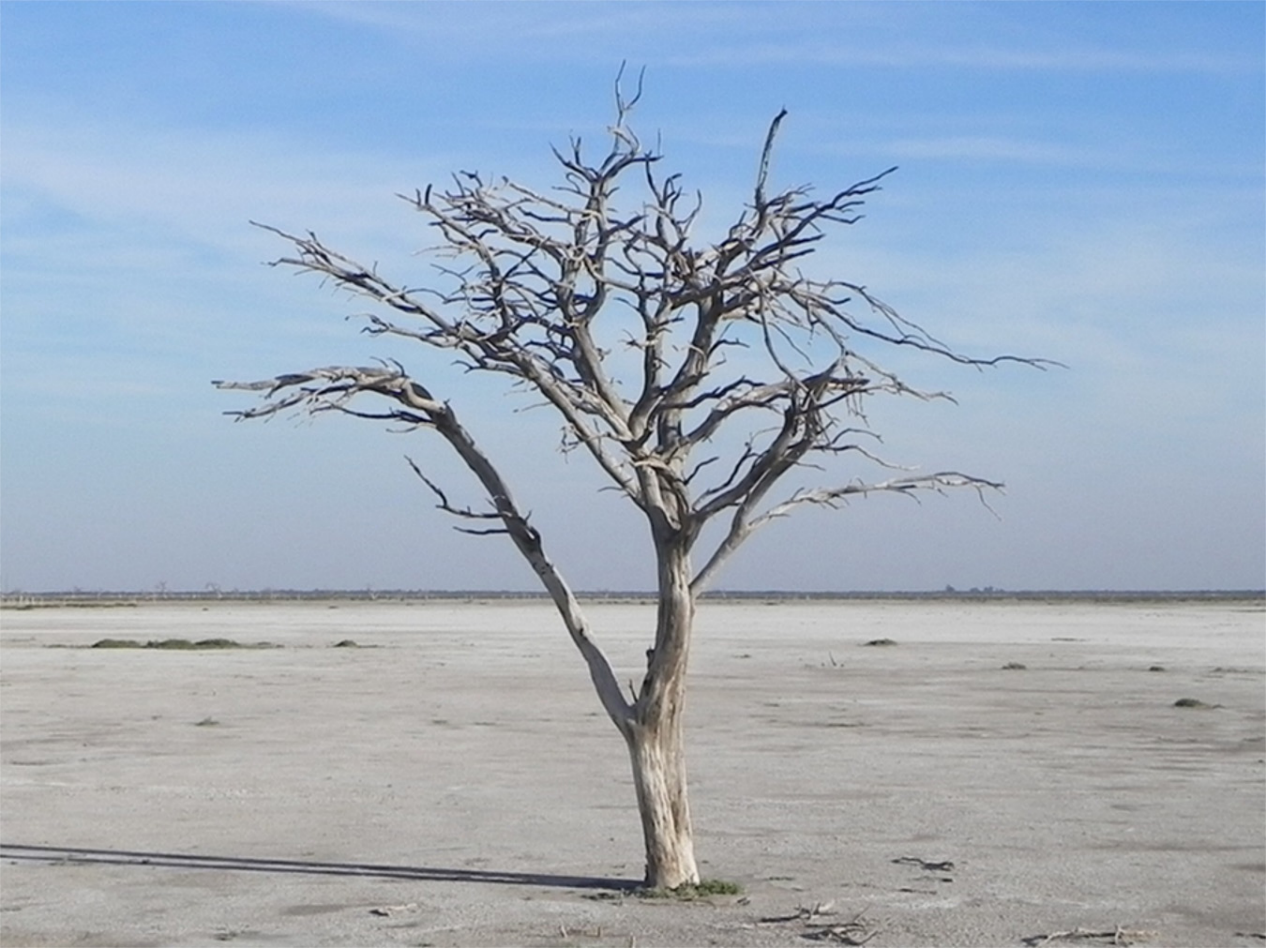
Dead tree from the original forest that surrounded the lake. The forest disappeared during the exceptional expansion during the 1972–2004 period.

The fourth period (2003–2013), was characterized by a steady and significant decline in level that went again below the 68 m a.s.l. level, closing a cycle of about 41 years since the start of the high water period (1972–2013) [7]. The lake area shrunk from a maximum of 7319 km^2^ in 2003 to 2448 km^2^ in 2014, leaving about 4871 km^2^ of exposed dry bottom mudflats (Figs 3, 4).

### Use of MODIS images

Occurrence of SDS in Mar Chiquita was monitored during the study period using NASA MODIS (Moderate Resolucion Imaging Spectroradiometer) images, AERONET Cordoba-CETT Subsets, obtained from the WEB page https://lance.modaps.eosdis.nasa.gov. SDS events were observed in the AERONET Cordoba-CETT subsets. We used 250 m pixel size images, from both Aqua and Terra satellites, and in true color (all frequencies) or filtered in the 7,2,1, band combination. Images were checked daily throughout the study period, unless cloud cover precluded ground observation.

We defined a SDS event as a salt dust plume originated from the lake area that in the MODIS image had at least 10 km in length and was more than one km wide. For our statistics, we considered as one SDS day when at least one SDS were recorded on the same date.

Wind speed on the coast of Mar Chiquita on days when salt dust events occurred was obtained from a Davis Vantage Vue Weather Stations (Scientific Sales, Inc. Lawrence, NJ, USA) near the southern coast of the lake (coordinates: 30.9555556° S, 62.8250000° W) (S1 Table). We also collected soil samples from the salt-dust generating areas. Soil samples collection was carried out within the boundaries of the Mar Chiquita and Bañados del Rio Dulce Reserve, province of Córdoba.

### Dust dispersion model

We estimated the atmospheric transport and dispersion of the salt storms using HYSPLIT ([10, 11]), a complete modeling system widely used in numerous applications dealing with the emission, transport, dispersion, and deposition of dust over different parts of the globe (e.g. [10, 11, 12, 13, 14, 15, 16, 17]. The spatial distribution of the deposited material reflects the integral effect of the emissions, transport, dispersion, and deposition throughout the year [18].

We defined the potential dust source locations by month, based upon a 5-year climatology model constructed from the MODIS Deep-Blue Aerosol Optical Depth’s (AOD) values [4] around the salt lake area. According to the approach developed by [12], dust emissions are presumed to occur at locations where the friction velocity (U*), calculated from the National Center’s for Environmental Prediction (NCEP)/ Global Data Assimilation System (GDAS) meteorological data [19], exceeds a threshold friction velocity (U*t). The U*t is computed from the AOD climatology at each location assuming that the probability that U* will exceed that value is the same as the probability that the AOD will exceed 0.25. Each emission grid point has a different Probability Density Function and, therefore, has a unique U*t. Furthermore, the emission rate (F) for a particular location is estimated by the expression

F=K(U*−U*t)A, (1)

where K is an empirical factor (for details see [12]) and A the area of the dust sources. Because of the empirical nature of this approach, K and U*t also contain the effect of all other physical processes not explicitly incorporated into the emission model. The threshold velocities defined at the emitting grid cells vary by month, which at least should capture the annual cycle in soil moisture and vegetation cover. Also, the spatial variation in U*t may reflect differences in soil moisture and land cover. No attempt was made to consider dynamic corrections to the emissions due to variations in soil moisture, except for shutting them off when the meteorological model indicated it was raining.

Every model time step, 3-dimensional lagrangian particles are emitted from grid cells where the friction velocity from the meteorological data exceeds the threshold friction velocity at that grid cell. The total mass of all particles emitted over that hour equals K(U-U*t)A. After the emissions take place, the model calculates the advection, dispersion, and wet and dry deposition of the 3D lagrangian particles over a 0.1x0.1 degree grid spanning over 60^0^ latitude and 40^0^ longitude centered over the lake. Salt dust particles are assumed to be spherical, with an average diameter of 4 μm, similar to a desert dust storm [6, 12, 17]. Emitted particles will gravitationally settle and can be removed by rainfall. HYSPLIT runs are driven by the NCEP/GDAS meteorological data [19] with a 1x1 degree spatial resolution and a 3-hour temporal resolution.

## Results

### Salt dust storm sources and characteristics

**Fig 5:**
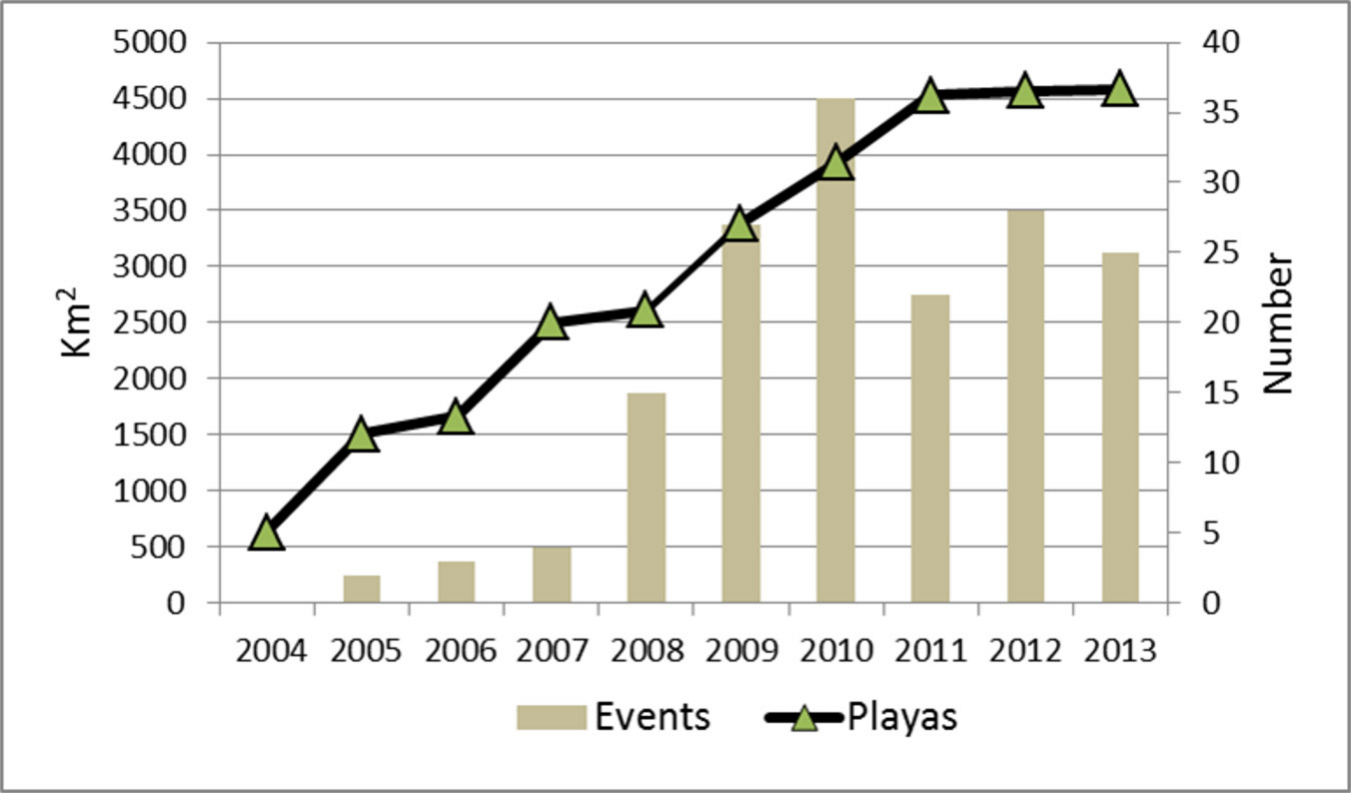
Area occupied by salt playas and number of salt dust events recorded in Mar Chiquita during the study period using MODIS images.

Large, previously unseen SDSs were observed for the first time in July 2004 [4].They originated exclusively from the wide area of salt playas left by the receding waters of the lake (Fig 4). This area started to expand in 2004, reaching 4500 km^2^ in 2011, and then remained relatively stable until the end of the study period (Fig 5).

**Fig 6:**
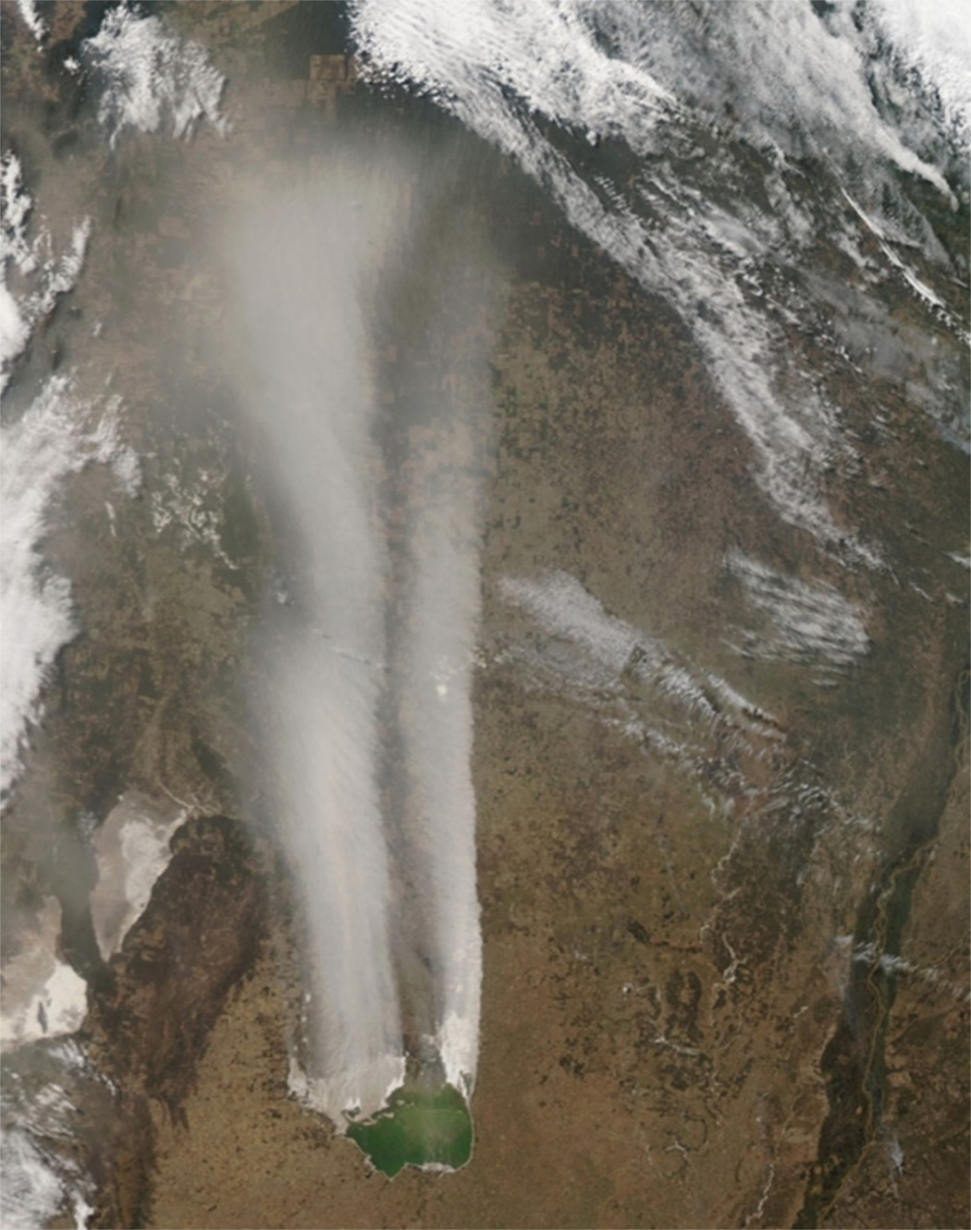
MODIS image of an exceptionally strong salt dust event generated in Mar Chiquita. Date: September 10, 2013. Distance reached by the visible plume: about 800 km. Modis Image: AERONET Cordoba-CETT (aqua), 250 m pixel size, full color.

Source points from where dust storms originated fluctuated from specific, limited areas of variable size to the whole salt flat area on days of strong winds and favorable soil conditions. The extension of the dust plumes ranged between about 50 and 800 km. An example of an exceptional magnitude SDS is shown in Fig 6.

Annual frequency of SDS events correlated with the size of the salt mudflats left by the receding waters of the lake (Fig 5). The number of events remained below five until 2008, and then increased markedly, peaking in 2010 with 36 events. From 2011 to 2013, annual SDS events ranged between 25 and 28 (Fig 5).

**Fig 7:**
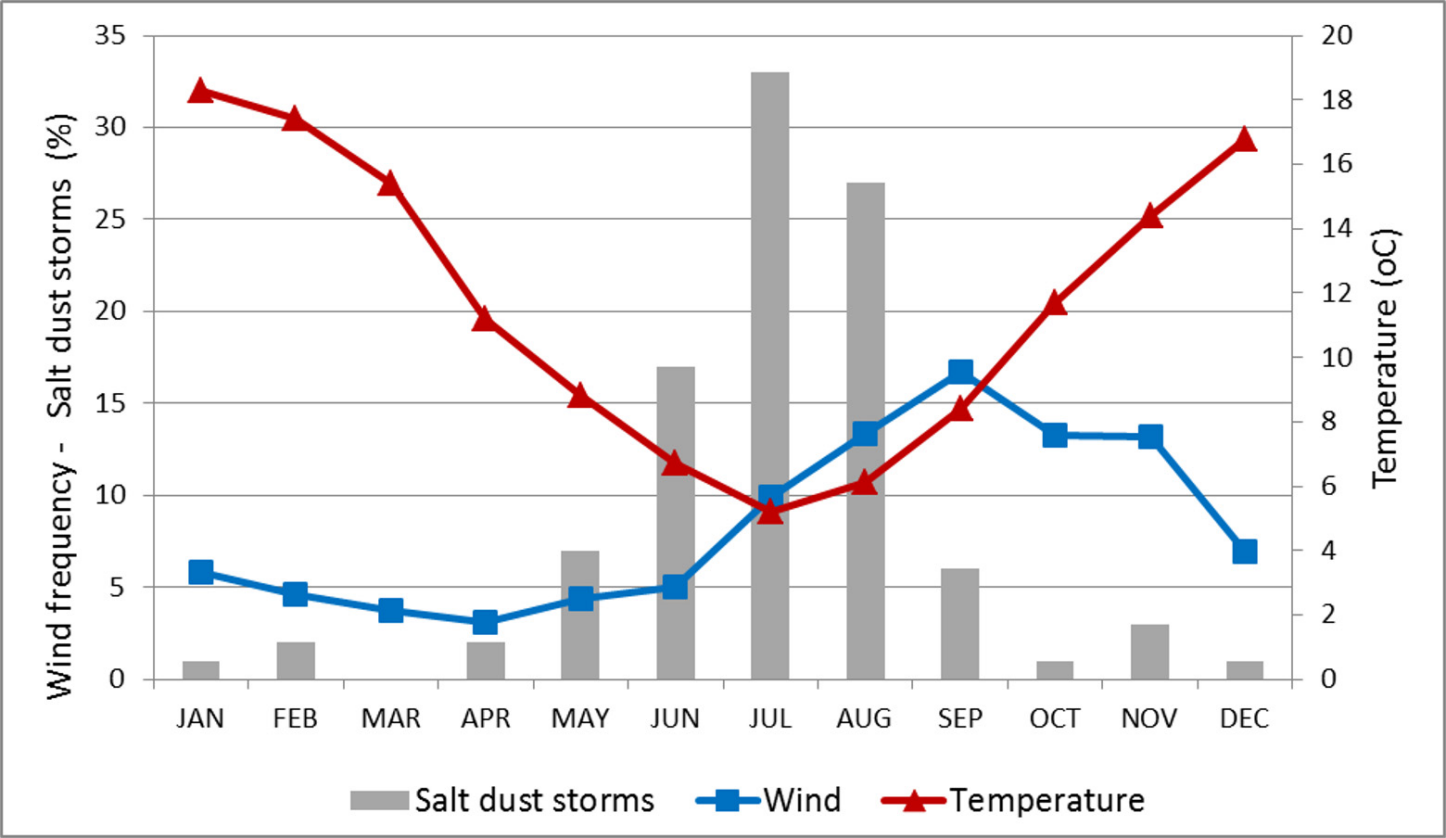
Mar Chiquita: monthly frequency of salt dust storms (%), days with wind over 27 km/h (%), and mean monthly minimal temperature (C). Temperature records from Miramar, 30° 57’S; 56° 20’ W. Servicio Meteorológico Nacional, Argentina.

Seasonal frequency of SDS events (Fig 7) indicated an almost complete restriction to the cold months of the year (May-September), with a clear peak in the coldest months (June, July, and August). Mean monthly temperature during the May-September period was below 10°C.

### The wind factor

Wind speeds were extracted from the NCEP/GDAS meteorological data for a representative location (30.75 S, 62.5 W). Analysis of these data indicated that the SDS occurred when the wind speed exceeded 7.5 m/s. The most intense salt dust storms were associated with stronger winds. However, wind intensity was not a dominant factor in the generation of salt dust storms. Instead, wind intensity appears to be a necessary, but not sufficient condition for the development of salt dust events. On similar windy days, SDS were more likely to occur in winter than in summer months.

As shown in Fig 7, the seasonal pattern in wind intensity did not overlap entirely with the annual distribution of SDS. Months of high frequency of winds above 7.5 m/s threshold include the July-November period, whereas months with high frequency of SDS occurred only in the June-August period.

### Substrate emissivity and MODIS images

**Fig 8:**
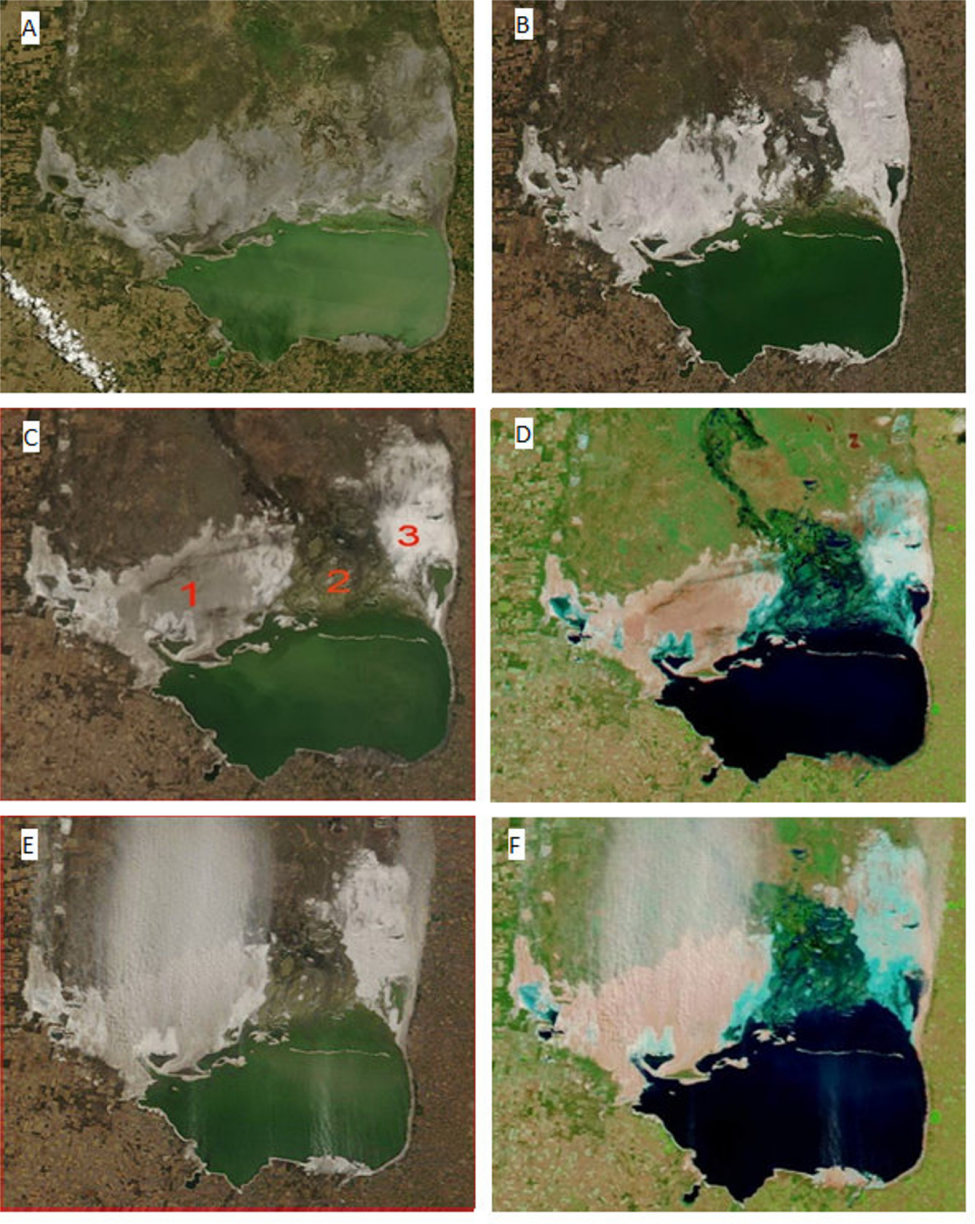
Stages in Mar Chiquita mudflat playas according to emission condition. A: gray; B: white; C: mosaic of surface patterns under dry conditions cold conditions: 1) Gray, 2) Striped brown (wetlands of the Dulce River delta); 3) Bright white; D: same image, 721 bands; E: and F: same as in C and D, eight days later. Notice the intense salt dust emission from the gray surfaces. MODIS images (AERONET_Cordoba-CETT), 250 m resolution, true color (unless 721 bands are indicated): A: January 21, 2014 (terra); B: June 25, 2013 (terra); C: July 16, 2012 (aqua); D: July 16, 2012, 721 bands (terra); E: July 24, 2012 (terra), F: July 24, 2012, 721 bands (terra).

Our observations indicate a clear association between ground condition (as shown in MODIS images) and SDS emission. By comparing a daily sequence of images for at least ten days before a SDS event, it was possible to identify different color patterns, their temporal variation, and their association with specific areas from where SDS were generated (Fig 8).

From the MODIS images, three color patterns are clearly distinguishable (Fig 8). In true color, we named them: Striped brown (Fig 8C2), Gray (Fig 8C1), and Bright white (Fig 8C3). In the MODIS 721 bands, the Striped brown areas appear greenish, the Gray areas pale brown, and the Bright white areas blue (Fig 8D).

The Striped brown pattern indicates recent heavy rains or areas with a surface water sheet (as the Dulce River delta). After heavy rains, the whole mudflat area may show this pattern for some time, until the surface area becomes drier (Fig 8C2).

The drying striped pattern usually turns into gray (Fig 8C1). Field observations indicate that this pattern may correspond to areas with a thick crust with a dominance of sodium chloride (NaCl). Very few cases of salt dust emission were observed originating from this substrate.

The Bright white pattern appeared mostly in cold weather, usually following rains, occasionally covering the whole mudflat area, but more commonly developed as patches closer to wetter areas such as the lake coast (Fig 8C3). The bright white pattern may turn into a gray substrate under dry conditions (somewhat darker than the previously mentioned Gray pattern), and back to Bright white in presence of rain or shallow flooding. This gray substrate is highly erodible and from it originated most of the observed salt dust events (Fig 8C1, 8E, and 8F).

The whole cycle of bright white appearance, transformation into gray substrate and development of dust storm events may be very short, from a minimum of three days to an average of about a week, apparently depending on the temperature and wind strength. Moreover, this cycle may occur several times in the same area throughout the cold weather season under adequate conditions, particularly cycles of wet-dry conditions and sufficiently strong winds.

### Dispersion model

HYSPLIT was run for the entire year 2009 to simulate the spatial and temporal evolution of the emitted salt dust. The empirically based algorithm used to estimate emissions [12] indicates that the area adjacent to the lake becomes a potential source of dust only from May to September. And as shown in Fig 7, most of the observed dust events occur over those months.

**Fig 9:**
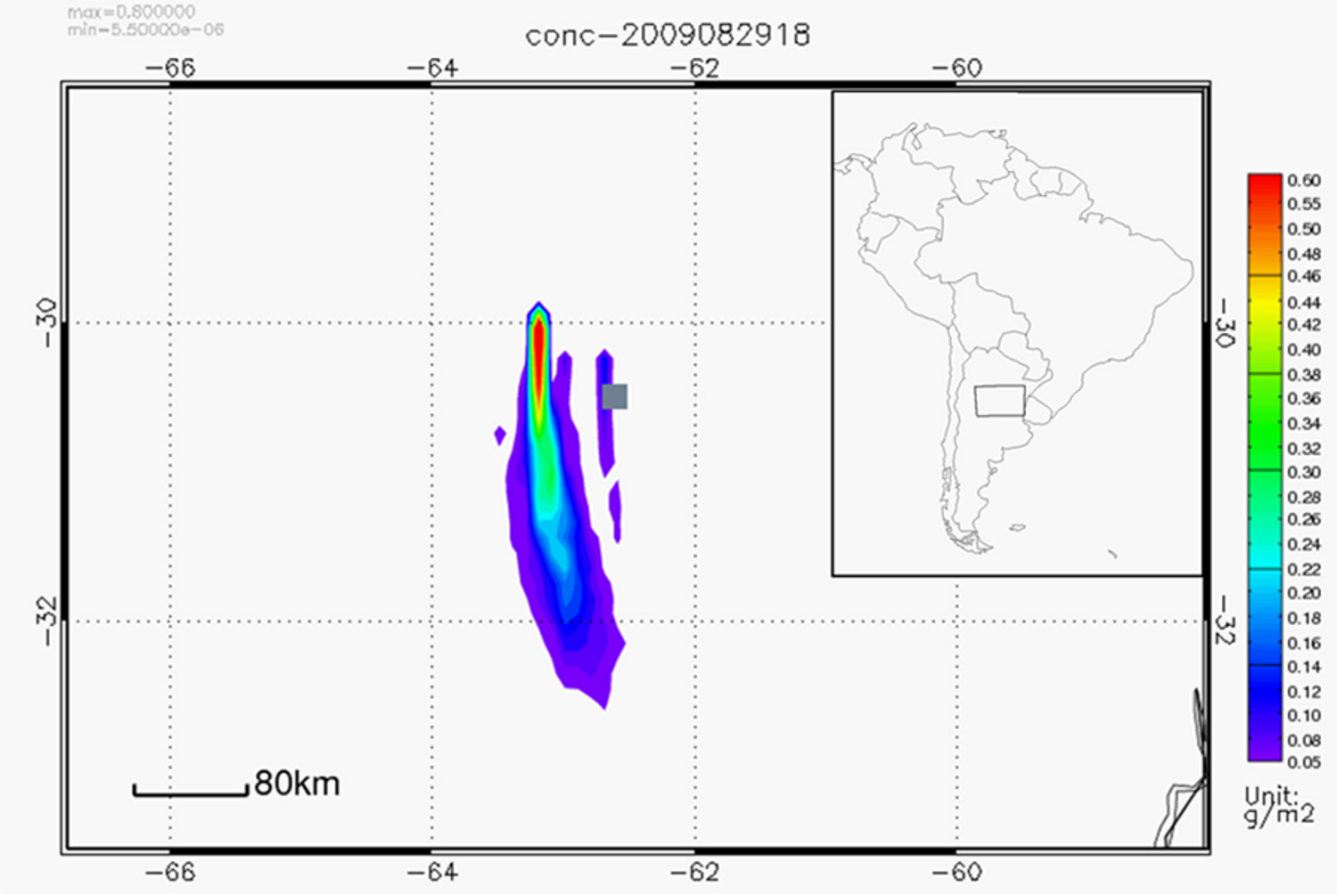
Simulated salt dust integrated column [g m^-2^] generated in Mar Chiquita on August 29, 2009 at 18:00 UTC. The grey square represents the location of center of the Mar Chiquita Lake.

**Fig 10:**
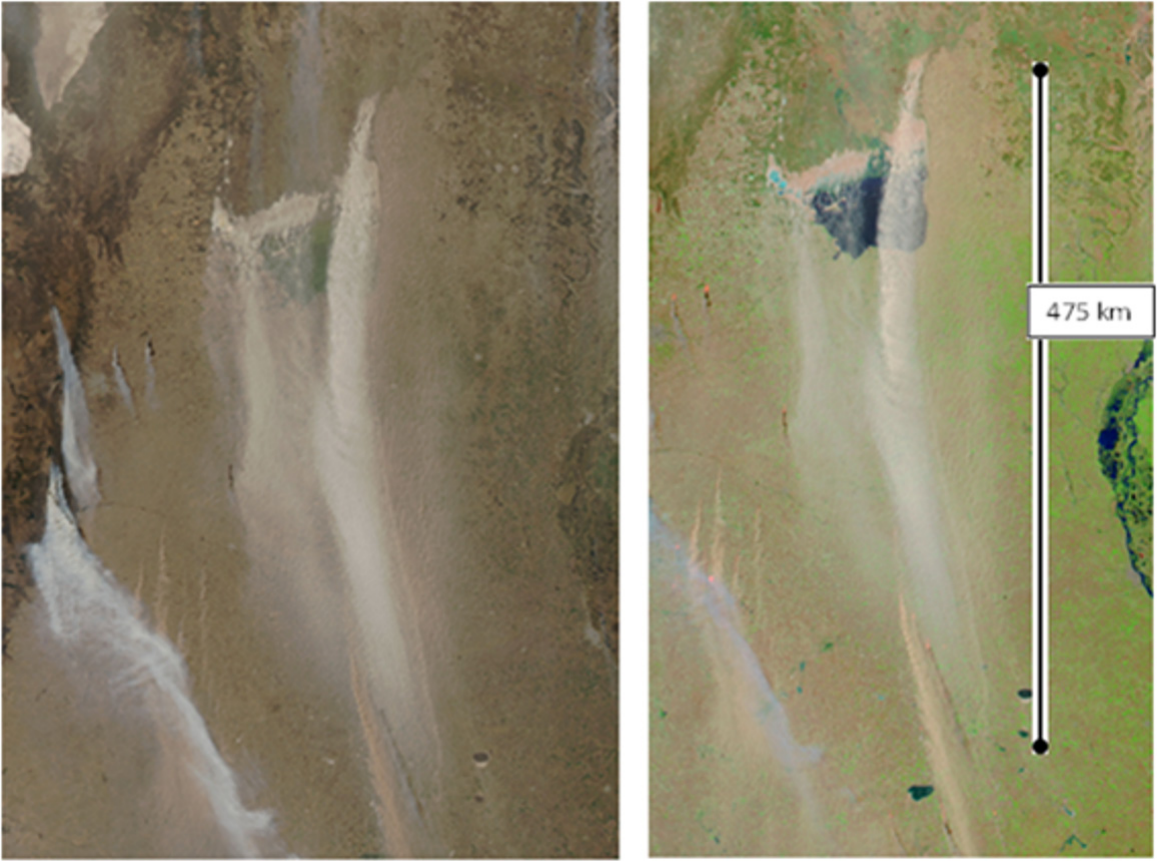
MODIS image of a salt dust event in Mar Chiquita on August 29, 2009. Left, true color, right, 721 bands. Image: MODIS AERONET Cordoba-CETT, aqua, 250 m pixel resolution.

Fig 9 shows an example of the HYSPLIT simulated column of integrated concentration of salt dust material. In this case, the strong northerly wind carries particles to the south. For a qualitative comparison, Fig 10 shows the satellite image corresponding to the same time period.

**Fig 11:**
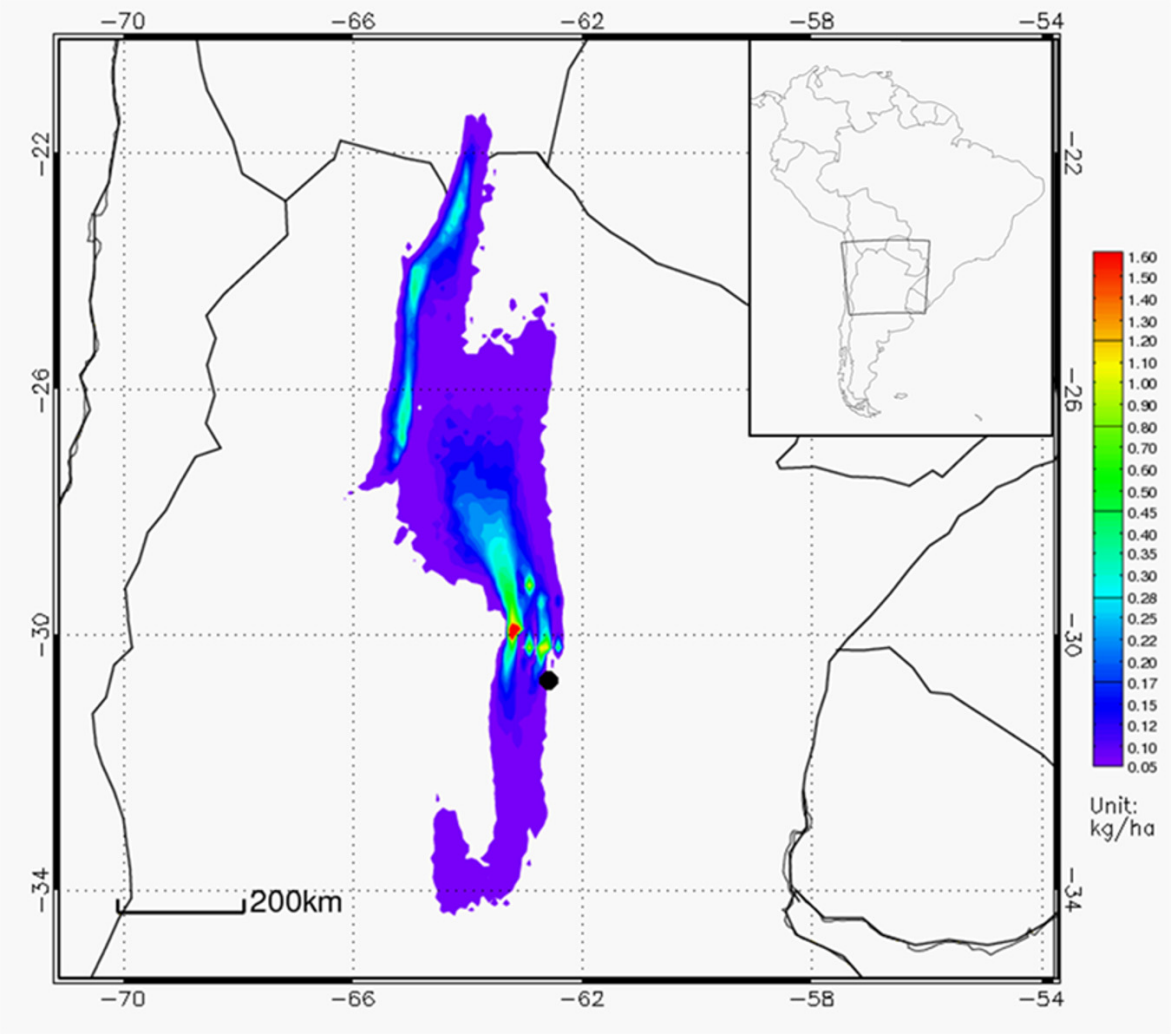
Model estimated annual (2009) dust deposition (kg/ha). The black dot represents the center of the Mar Chiquita Lake.

The annual (2009) simulation shows (Fig 11) a deposition maximum near the sources (of about 2.5 kg/ha/yr), and a decreasing trend from the emission area, except for the relative secondary maximum modeled over the mountain ranges in southern Bolivia and northern Argentina due to their orographic effect (Fig 11). The deposition pattern reflects predominance of the northerly and southerly wind components, typical of the local climatic conditions. The 2009 total deposition of salt dust generated in Mar Chiquita was estimated in 6.5 million tons. Note that this estimate will strongly depend on the underlying assumptions about the shape and size of the deposited particles, which for this work were assumed to be similar to those emitted by a dust storm [14, 16, 17, 18]. Further characterization of the emitted salt particles is necessary to provide a more accurate estimate of the total deposition.

## Discussion

### Mar Chiquita, a special case of a salt-dust emitting lake

The recent development of intense SDS events in Mar Chiquita originated from a climatic cycle with two sequential and contrasting events. First, a 20-year-long period of above average rainfall in the lake basin that produced a three-fold increase of the lake area expanding in areas previously occupied by stable vegetation and never flooded before along the lake’s geological history, followed by a ten-year, low-rain period that resulted in water retreating and the development of vast salt playas. Therefore, the Mar Chiquita case should be taken into consideration in terms of its implications for the understanding of climate change effects on South American ecosystems. In terms of its potential impact, the annual deposition produced by Mar Chiquita, estimated at about the 6.5 million tons for the year 2009, is smaller than the 43 million tons found for the Aral sea [4], although still significant and proportionally similar if we consider the smaller size of Mar Chiquita.

Specifically, the case of Mar Chiquita deserves at least two important considerations. In the first place, it implies a significant, although little understood factor in the salt balance of the lake, with clear hydrological and limnological implications [6]. Secondly, more research is needed regarding the potential impact of salt dust deposition in an area where the predominant land use includes non-irrigated agriculture and cattle raising, associated with a relatively high human population density.

### MODIS images and salt playas emissivity characteristics

The observed correlation between MODIS image characteristics and salt erodibility of Mar Chiquita has an interesting potential for the assessment and prediction of salt dust storms in the lake. More specifically, our results show that the changing mudflat color patterns provide interesting information about the ground surface conditions in terms of salt dust erodibility.

Based on the high proportion of sulfates in Mar Chiquita water and also in the playas evapoprites, second only to chlorine [20], we hypothesize that the observed color patterns are compatible with different degrees of predominance of the mirabilite-thenardite component (hydrated/anhydrate forms of sodium sulfate) in the evaporite salts of the mudflats. Therefore, the Bright white pattern may be associated with predominance of the highly reflective mirabilite crystals.

This hypotheses is supported by the fact that salt crusts composed of hydrous/anhydrous minerals (such as mirabilite/thenardite), are more likely to dissolve and re-precipitate repeatedly (i.e., diurnally or seasonally), generating loose crusts that result in highly reflective surfaces [21]. An important pre-condition for the development of this reflective surface is the existence of a shallow depth of ground water, which was predominant in Mar Chiquita salt playas during the study period. Therefore, the Gray surface that usually follows the Bright white pattern may be assigned to a characteristic “fluffy” surface, which is composed of a mixture of less reflective evaporites (including thenardite as a significant component) and silicate minerals derived from the salt precipitation–dissolution processes [21].

The fact that the White bright pattern is relatively short-lived and occurs almost entirely during winter months also follows the mirabilite property of crystalizing under low temperatures and rapidly dehydrating and turning into thenardite when exposed to air warmer than its transition temperature [21]

Further evidence supporting the predominance of the mirabilite\thenardite deposits in Mar Chiquita comes from reports that in the early 20^th^ century salt deposits along the Mar Chiquita shoreline were common but differed markedly between seasons. In summer, sodium chloride was predominant (94.8%) and sodium sulfate (Glauber salt) was not detected, whereas in winter sodium sulfate was dominant (95.7%) and sodium chloride was reduced to only 1.5% [20]. Higher frequency of salt dust emissions in winter has also been recorded in other lakes, particularly in the Mojave Desert in California [22] and the Salton Sea [23] and; in both cases the surface crust of evaporites was dominated by the mirabilite/thenardite system.

## Conclusions

- Unlike what is commonly observed in salt lakes, the recent development of vast salt playas with high SDS occurrence in Mar Chiquita was not the caused by water withdrawal. Instead, it derived from a 30-year cycle of exceptional rainfall that produced a three-fold expansion and retreat of the lake area.
- SDS occurrence shows a clear concentration in the winter, which is consistent with the predominance of hydrated/anhydrated forms of sodium sulfate in the evaporite salts of the salt playas
- MODIS images provide a useful tool for the understanding the temporal and spatial heterogeneity and dynamic of the salt playas, with great potential for the understanding and prediction of salt dust generation in salt lakes.

## Supporting Information

S1 Table Mar Chiquita Wind. Wind speed components (N-S, E-W) [m/s] and temperature [K] extracted from the NCEP/GDAS meteorological data for the location -30.75 S, -62.5 W.
(TXT)
